# Chemopreventive Effect of *Aster glehni* on Inflammation-Induced Colorectal Carcinogenesis in Mice

**DOI:** 10.3390/nu10020202

**Published:** 2018-02-12

**Authors:** Kyung-Sook Chung, Se-Yun Cheon, Seong-Soo Roh, Minho Lee, Hyo-Jin An

**Affiliations:** 1 Catholic Precision Medicine Research Center, College of Medicine, The Catholic University of Korea, 222, Banpo-daero, Seocho-gu, Seoul 06591, Korea; adella76@hanmail.net; 2 Department of Pharmacology, College of Korean Medicine, Sangji University, 83 Sangjidae-gil, Wonju-si, Gangwon-do 220-702, Korea; chunsay1008@naver.com; 3 Department of Herbology, Daegu Haany University, Daegu 42158, Korea; ddede@dhu.ac.kr

**Keywords:** *Aster glehni*, colitis-associated cancer, inflammation, NF-κB, chemoprevention

## Abstract

Although *Aster glehni* is a common dietary herb that has various bioactivities, including anti-diabetic, anti-adipogenic, and anti-inflammatory effects, *A. glehni* has not been studied in colon cancer. Therefore, we hypothesized the chemopreventive effects of an ethanol extract of *A. glehni* (AG) on azoxymethane/dextran sulfate sodium (AOM/DSS)-induced colitis-associated cancer (CAC) in mice. In this study, we found that treatment with AG significantly attenuated the AOM/DSS-induced enlargement of the spleen and shortening of the colon. In addition, colonic tumor formation, colonic damage, and increased muscle thickness were significantly reduced in AOM/DSS-induced mice fed AG. Treatment with AG also reduced intestinal interleukin (IL)-1β, IL-6, and tumor necrosis factor (TNF)-α production and decreased inducible nitric oxide synthase (iNOS) and cyclooxygenase (COX)-2 protein expression in mice with AOM/DSS-induced CAC. Furthermore, AG reduced nuclear factor (NF)-κB activation via phosphorylation and degradation of inhibitor of kappa Bα (IκBα), leading to inhibition of NF-κB p65 nuclear translocation. It also downregulated the expression of NF-κB-related proteins, including the B-cell lymphoma 2 (Bcl-2) family and inhibitors of apoptosis proteins (IAPs), in mice with AOM/DSS-induced CAC. Taken together, these findings suggest that the treatment with AG inhibited colitis-associated colon carcinogenesis in mice, and this chemopreventive effect was strongly mediated by suppression of the NF-κB signaling pathway, indicating that AG could be a promising protective agent against CAC.

## 1. Introduction

Inflammatory bowel disease (IBD) refers to a group of chronic dysregulated inflammatory conditions in the large and small intestine of humans, and it is well known that chronic inflammation in the colon can lead to cancer [1]. Colitis-associated cancer (CAC), the colorectal cancer (CRC) subtype that is associated with IBD, is difficult to treat, and has a high mortality rate [2]. More than 20% of IBD patients develop CAC within 30 years of disease onset, and >50% of them will die from CAC [3]. CRC is caused by the accumulation of mutations in oncogenes and tumor suppressor genes. Mutations in the adenomatous polyposis coli (APC) oncogene-related pathway mediate the transition of single preneoplastic cells to aberrant crypt foci (ACF), and then to adenoma and colorectal carcinoma [4]. Chronic inflammation, which leads to CAC, is characterized by the production of pro-inflammatory cytokines. These can induce mutations in oncogenes and tumor suppressor genes, and cause genomic instability via various mechanisms. Persistent inflammation facilitates tumor promotion by altering tumor response to chemotherapeutic drugs, and activating proliferation, tumor progression, and metastasis [5]. Accumulating evidence has shown that certain dietary agents have anti-inflammatory and anti-carcinogenic effects, and they can be potentially utilized as chemopreventive agents against inflammation-associated colon carcinogenesis [6].

Many dietary natural compounds, such as flavonoids, polyphenols, carotenoids, and isothiocyanates in fruits and vegetables, have been isolated, and their health-promoting properties have been demonstrated [7]. Chemoprevention is defined as the use of natural dietary compounds and/or synthetic substances to delay, prevent, or even reverse the development of adenomas, as well as the progression from adenoma to carcinoma. The molecular mechanisms of their chemo-preventive action are associated with the modulation of signaling cascades and gene expressions involved in the regulation of cell proliferation, differentiation, and apoptosis and the suppression of chronic inflammation and metastasis. *Aster glehni* Franchet et Sckmidt is a traditional edible herb, which is distributed on the Ulleung-do island of the Republic of Korea. Previous studies by our group have analyzed caffeoylquinic acids (CQs), such as 3,5-di-*O*-dicaffeoylquinic acid, 5-*O*-caffeoylquinicacid, 3-*O*-caffeoylquinic acid and 3-*O*-*p*-coumaroylquinicacid, and flavonoids, such as astragalin and kaempferol, in *A. glehni* using high-performance liquid chromatography (HPLC) [8]. In addition, we also observed that the ethanol extract of *A. glehni* has anti-adipogenic effects on obesity, including the downregulation of adipogenesis-related transcription factors, and has anti-inflammatory effects which are mainly related to the inhibition of the expressions of inflammatory mediators via NF-κB inactivation in mice with DSS-induced colitis. These findings indicate that the intake of *A. glehni* may result in enhanced protection from the development of CAC commonly associated with IBD. In the present study, we aimed to evaluate the chemopreventive effects of *A. glehni* on a mouse model of azoxymethane (AOM)/dextran sulfate sodium (DSS)-induced colitis-associated tumorigenesis, by characterizing several inflammatory markers and the key pathways involved.

## 2. Materials and Methods

### 2.1. Materials

DSS was purchased from MP Biomedicals (Santa Ana, CA, USA). IL-1β, IL-6, and TNF-α enzyme immunoassay (EIA) kits were purchased from R&D systems (Minneapolis, MN, USA). Primary antibodies for iNOS (M-19), COX-2 (C-20), p-IκBα, IκBα (H-4), p65 (A), B-cell lymphoma-2 (Bcl-2; C-2), XIAP (D-2), survivin (D-8), β-actin (ACTBD11B7), α-tubulin (TU-02), and nucleolin (C23) and H-6 primary antibodies were purchased from Santa Cruz Biotechnology, Inc. (Dallas, TX, USA). Peroxidase-conjugated secondary antibodies were purchased from Jackson ImmunoResearch, Inc. (West Grove, PA, USA). AOM, 5-aminosalicylic acid (5-ASA), hematoxylin and eosin (H&E), and all other chemicals were purchased from Sigma-Aldrich Co. (St. Louis, MO, USA).

### 2.2. Preparation and Standardization of the Extract of A. glehni

*A. glehni* (AG) extracts were prepared as described previously [9]. The dried material was refluxed with 70% EtOH for 6 h at 60 °C. The extract was filtered (Whatman Qualitative Filter Paper No. 4, 20–25 μm, GE Healthcare Life Sciences, Seoul, Korea), concentrated under reduced pressure, and then freeze-dried (−50 °C, under pressures between 20 and 30 Pa) to obtain a solid extract powder (73 g). HPLC analysis was performed using a Gilson system equipped with a 234 autosampler, a UV/VIS-155 detector (Gilson, Seoul, Korea), and a Luna 4.60 × 250 mm C18 reversed-phase column with 5 µm particles (Phenomenex, CA, USA). Chromatography was performed at 25 °C with a flow rate of 0.5 mL/min, and 10 µL was analyzed for 50 min. CQs and AG dissolved in 80% MeOH were filtered using a syringe filter and injected for HPLC analysis. The two mobile phases of 0.05% phosphoric acid (solvent A) and MeOH (solvent B) were used for gradient elution at the rate of 1.00 mL/min: 0–10 min, 60% A:40% B; 10–20 min, 50% A:50% B; 20–30 min, 40% A:60% B; and 30–35 min, 60% A:40% B. The two flavonoids, astragalin and kaempferol, were also used as the standard compounds for the analysis. Sample solutions were injected into the HPLC system at 1.000 mg/mL, and the contents were determined from the regression equation. The concentrations of four CQs (3,5-DQ, 5-CQ, 3-CQ, and 3-pCQ) and two flavonoids (astragalin and kaempferol) were determined to be 4.43, 13.69, 3.25, 34.06, 3.2, and 0.23 mg/g, respectively.

### 2.3. Experimental Animals

All the animal experiments were conducted under university guidelines and approved by the Animal Care Committee of Sangji University (Animal Experimental Registration Code: 2014-13, Wonju-si, Korea). A total of 40 male C57BL/6 mice (6 weeks of age) were obtained from Daehan Biolink (Eumsung, Korea). During the experimental period, the mice were kept in cages, in groups of ten, and maintained in air-conditioned quarters with a constant temperature (temperature, 20 ± 5 °C; humidity, 40–60%) and an alternating 12 h dark/light cycle.

### 2.4. AOM/DSS-Induced CAC Model and Treatment

The AOM/DSS method has been established to induce inflammation-driven CRC [10]. For the experiment, the animals were divided into 4 groups (*n* = 10/group): the control group (normal colon treated with vehicle solution: 5% EtOH and 5% Cremophor in saline, p.o.); the AOM/DSS group (AOM/DSS-induced CAC group treated with vehicle solution, p.o.); the 5-ASA group (AOM/DSS-induced CAC group treated with 5-ASA 75 mg/kg/day; p.o.); and the AG group (AOM/DSS-induced CAC group treated with AG 25 mg/kg/day, p.o.).

**Figure 1:**
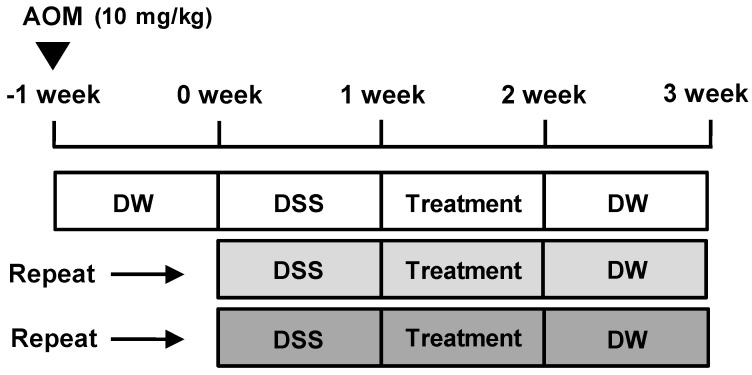
Schema of the AOM/DSS-induced mice model. AOM/DSS-induced mice were treated with 5-ASA (75 mg/kg/day; i.p.) or AG (25 mg/kg/day; p.o.) as described in Materials and Methods. 5-ASA was used as a reference substance. DW = distilled water

Except for animals in the control group, all animals were injected intraperitoneally with AOM in PBS (10 mg/kg) to induce AOM/DSS-induced CAC model (Figure 1). One week later, the AOM-treated mice were administered a course of 2% DSS in drinking water ad libitum for 1 week. After the cessation of DSS administration, control group and AOM/DSS group animals daily received vehicle solution, and 5-ASA group and AG group animals daily received each treatment (75 mg/kg/day and 25 mg/kg/day, respectively) for 1 week. After treatment duration, all animals were provided drinking water ad libitum for 1 week without any treatment and this course was repeated 3 times to induce CAC animal model. Mice were killed on the last day by cervical dislocation, and their colons and spleens were removed and colons stained with 0.2% methylene blue to determine number of macroscopic tumors.

### 2.5. Histopathological Analysis

The colon tissues were fixed with 10% paraformaldehyde and embedded in paraffin. For histopathological analysis, tissue samples were sectioned (5 µm) and stained with H&E to observe the extent of the severity of mucosal injury and crypt damage. The stained slides were observed with a Leica microscope (Leica DFC 295, Wetzlar, Germany) and photographed (Leica Application Suite, version 4.8.0, Heerbrugg, Aargau, Switzerland) [8].

### 2.6. Measurement of Cytokine Production

Colon tissues were prepared with homogenization with the Pro-prep^TM^ buffer (Intron biotechnology Inc, Kyungki-Do, Korea). Tissue debris was removed by microcentrifugation, followed by quick freezing of the supernatants. The protein concentration was determined using the Bio-Rad protein assay reagent according to the manufacturer’s instructions. The same concentration of proteins in colon tissues were used to measure cytokine levels. The levels of IL-1β, IL-6, and TNF-α in the proteins of colon tissues were quantified using EIA kits (R&D Systems, Minneapolis, MN, USA) according to the manufacturer’s instructions [8].

### 2.7. Western Blot Analysis

Same concentrations of protein samples were electrophoresed and transferred to polyvinylidene fluoride (PVDF) membrane. The immunoblotted membranes were incubated with primary antibodies in blocking solution (5% skim milk) overnight at 4 °C. Blotted membranes were washed three times with Tween 20/Tris-buffered saline (0.2% T/TBS) and incubated with a 1:2500 dilution of horseradish peroxidase (HRP)-conjugated secondary antibody for 2 h at room temperature. Blots were again washed three times with T/TBS, and then developed by enhanced chemiluminescence (GE healthcare, Milwaukee, WI, USA).

### 2.8. Nuclear Extraction

Colon tissues were resuspended in hypotonic buffer (10 mM HEPES, pH 7.9, 1.5 mM MgCl2, 10 mM KCl, 0.2 mM phenylmethylsulfonyl fluoride (PMSF), 0.5 mM dithiothreitol (DTT), 10 μg/mL aprotinin) and incubated on ice for 15 min. Cells were then lysed by adding 0.1% Nonidet P-40 and vortexed vigorously for 10 s. Nuclei were pelleted by centrifugation at 12,000× *g* for 1 min at 4 °C and resuspended in high salt buffer (20 mM HEPES, pH 7.9, 25% glycerol, 400 mM KCl, 1.5 mM MgCl_2_, 0.2 mM EDTA, 0.5 mM DTT, 1 mM NaF, 1 mM sodium orthovanadate).

### 2.9. Statistical Analyses

Results are expressed as mean ± S.D. of triplicate experiments. Statistically significant values were compared using ANOVA and Dunnett’s post hoc test, and *p*-values < 0.05 indicated statistical significance.

## 3. Results

### 3.1. AG Improved AOM/DSS-Induced Spleen Enlargement

**Figure 2:**
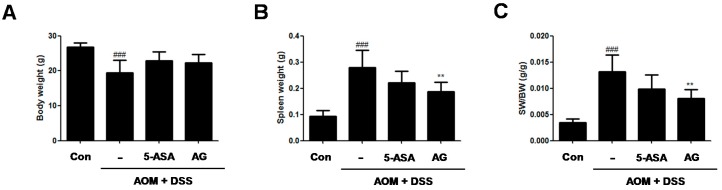
Effect of AG on body weight and spleen weight in AOM/DSS-induced mice. (**A**) Body weight; (**B**) spleen weight; and (**C**) spleen weight (SW)/body weight (BW) were measured at last day of AOM/DSS-treated experiments. Values represent mean ± S.D. of three independent experiments. ^###^ *p* < 0.001 vs. the control group; ** *p* < 0.01 vs. the AOM/DSS group; significances between treated groups were determined using ANOVA and Dunnett’s post hoc test. Con = Control.

To assess the chemopreventive effect of AG on the inflammation-mediated colon cancer, AOM/DSS-induced CAC animals was used. During the AOM/DSS-induced CAC experiment, no significant difference in the body weight between the AOM/DSS-treated group and the AG-treated group (Figure 2A) was found. As compared with vehicle-treated control mice, vehicle-treated mice with AOM/DSS exhibited spleen enlargement (Figure 2B, 0.09 ± 0.02 g vs. 0.28 ± 0.06 g, *p* < 0.001). 5-ASA and AG both reduced spleen sizes in mice with DSS-induced colitis; vehicle-treated mice with AOM/DSS versus 5-ASA (75 mg/kg)-treated mice with AOM/DSS (5-ASA 75 mg/kg: 0.28 ± 0.06 g vs. 0.22 ± 0.04 g, AG 25 mg/kg:0.28 ± 0.06 g vs. 0.19 ± 0.04 g, *p* < 0.01). Since AG treatment reduced the AOM/DSS-induced spleen enlargement, the ratio of SW/BW in the AG-treated group also significantly decreased compared to those of the AOM/DSS-treated group (Figure 2C).

### 3.2. AG Prevented AOM/DSS-Induced Colon Shortening and Tumor Formation

**Figure 3:**
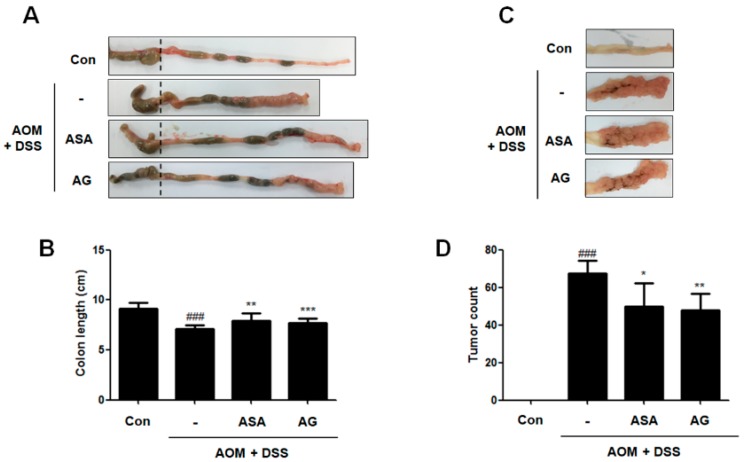
Inhibitory effect of AG on tumor progression in AOM/DSS-exposured mice. (**A**) Colons were obtained on the last day of AOM/DSS administration; and (**B**) colon lengths; and (**C**,**D**) number of macroscopic tumors were measured. Values represent mean ± S.D. of three independent experiments. ^###^ *p* < 0.001 vs. the control group; * *p* < 0.05, ** *p* < 0.01 and *** *p* < 0.001 vs. the AOM/DSS group; significances between treated groups were determined using ANOVA and Dunnett’s post hoc test.

DSS administration leads to shortened colons in mice [11]. As shown in Figure 3A,B, our data presented that the colon length of AOM/DSS-treated mice was significantly shorter than that of the control mice (9.13 ± 0.62 cm vs. 7.08 ± 0.36 cm, *p* < 0.001). In contrast, 5-ASA or AG treatment prevented the shortening of colon length caused by AOM/DSS; vehicle-treated mice with AOM/DSS versus 5-ASA (75 mg/kg)-treated mice with AOM/DSS (7.08 ± 0.36 cm vs. 7.93 ± 0.76 cm, *p* < 0.01); and vehicle-treated mice with AOM/DSS versus AG (25 mg/kg)-treated mice with AOM/DSS (7.08 ± 0.36 cm vs. 7.71 ± 0.41 cm, *p* < 0.001). Furthermore, we also observed that dietary AG decreased tumor formation in AOM/DSS-treated mice (Figure 3C,D); vehicle-treated mice with AOM/DSS versus 5-ASA (75 mg/kg)-treated mice with AOM/DSS (67.33 ± 7.02 vs. 49.75 ± 12.58 *p* < 0.05); and vehicle-treated mice with AOM/DSS versus AG (25 mg/kg)-treated mice with AOM/DSS (67.33 ± 7.02 vs. 47.80 ± 8.76, *p* < 0.01). Colon length and tumor formation in AG-treated mice were similar to those of 5-ASA-treated mice. These results indicated that treatment with AG alleviated the AOM/DSS-induced clinical signs and tumor development.

### 3.3. AG Ameliorated AOM/DSS-Induced Histopathological Signs and Thickness of Muscle Layer in the Colon

**Figure 4:**
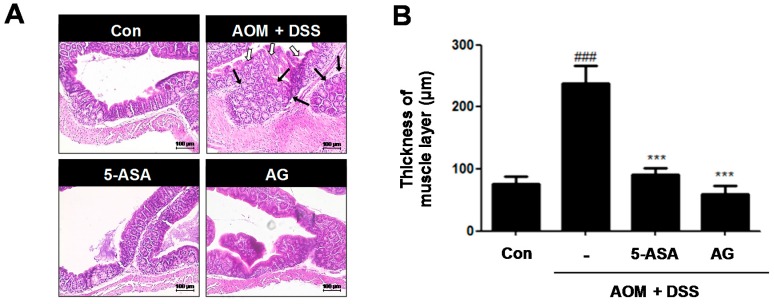
Effect of AG on analysis of colonic section in AOM/DSS-induced mice: (**A**) H&E staining of colonic section (Original Magnification × 100); and (**B**) thickness of muscle layer was evaluated using LAS software. Stained section was observed by microscope. Values represent mean ± S.D. of three independent experiments. ^###^ *p* < 0.001 vs. the control group; *** *p* < 0.001 vs. the AOM/DSS group; significances between treated groups were determined using ANOVA and Dunnett’s post hoc test.

Histological analysis confirmed that the AOM/DSS group had crypt destruction (white arrows) and even large adenocarcinomas (black arrows) in some specimens (Figure 4A). In contrast, 5-ASA or AG-treated groups indicated a marked reduction of these symptoms. It is reported that colonic smooth muscle thickness was used to estimate inflammation in the gastrointestinal tract [12]. As shown in Figure 4B, compared to the control group, the AOM/DSS-induced group revealed an increase of colonic muscle thickness (76.15 ± 12.21 μm vs. 237.85 ± 28.73 μm, *p* < 0.001). However, treatment with 5-ASA or AG significantly ameliorated the elevated muscle thickness caused by the AOM/DSS treatment; vehicle-treated mice with AOM/DSS versus 5-ASA (75 mg/kg)-treated mice with AOM/DSS (237.85 ± 28.73 μm vs. 90.77 ± 11.30 μm, *p* < 0.001) and vehicle-treated mice with AOM/DSS versus AG (25 mg/kg)-treated mice with AOM/DSS (237.85 ± 28.73 μm vs. 59.62 ± 13.14 μm, *p* < 0.001).

### 3.4. AG Attenuated the Production of Inflammatory Cytokines and the Expression of Inflammatory Proteins in AOM/DSS-Treated Mice

**Figure 5:**
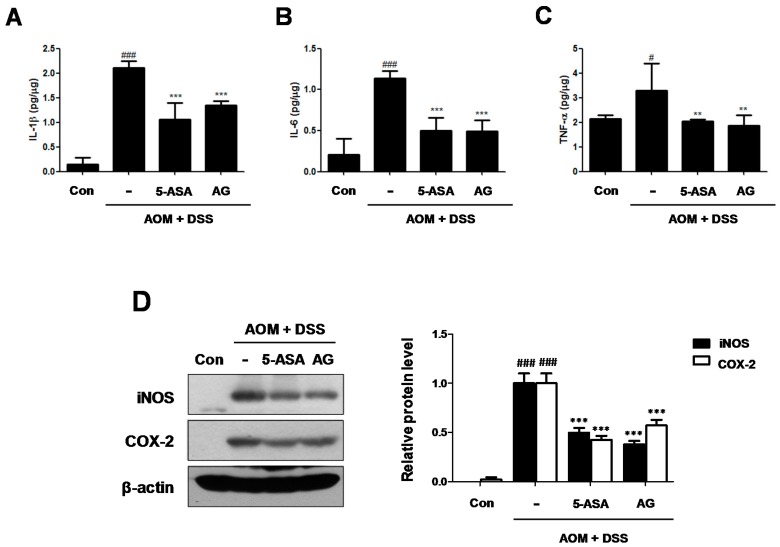
Effect of AG on the production of proinflammatory cytokines and the protein expressions in AOM/DSS-induced mice: (**A**) IL-1β; (**B**) IL-6 and (**C**) TNF-α were determined by EIA kits; and (**D**) Western blot analysis were performed for the protein expression level of iNOS and COX-2 using specific antibodies. Relative ratio level was determined by densitometric analysis (Bio-rad Quantity One^®^ Software) normalized to β-actin. Values represent mean ± S.D. of three independent experiments. ^#^ *p* < 0.05, ^###^ *p* < 0.001 vs. the control group; ** *p* < 0.01, *** *p* < 0.001 vs. the AOM/DSS group; significances between treated groups were determined using ANOVA and Dunnett’s post hoc test.

Cytokines play a major role in the immunopathogenesis of IBD and a crucial role in promoting neoplastic transformation [13]. To investigate the role of IL-1β, IL-6, and TNF-α in CAC, we assessed the production of these inflammatory mediators in AOM/DSS-induced colonic tissues. Although production of IL-1β, IL-6, and TNF-α were significantly increased in colonic tissue of the AOM/DSS group, treatment with 5-ASA or AG markedly suppressed the production of these inflammatory cytokines (Figure 5A–C).

In addition, the iNOS and COX-2 protein expressions in the colonic tissue were also determined (Figure 5D). Treatment with AOM/DSS significantly induced iNOS and COX-2 protein expression, whereas the expression of these proteins was downregulated by 5-ASA or AG treatment in the colon of mice.

### 3.5. AG Downregulated NF-κB Activation Involving NF-κB-Associated Protein Expression in AOM/DSS-Treated Mice

**Figure 6:**
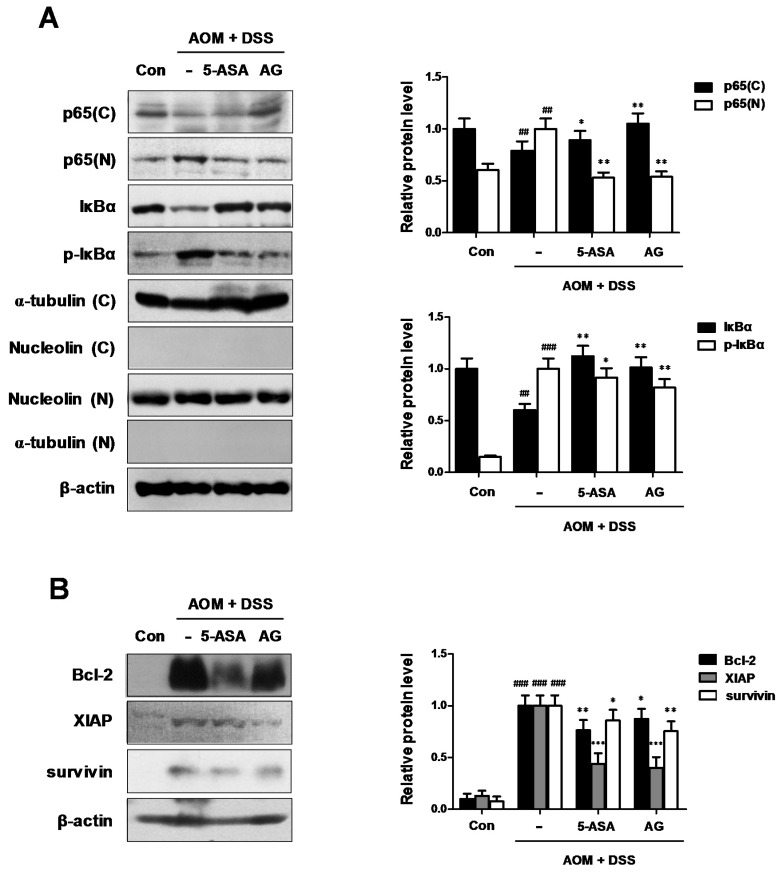
Role of AG on the NF-κB pathway in AOM-DSS-induced mice. (**A**) The nuclear translocation of p65 and expression of p-IκBα and IκBα were determined by Western blot analysis using specific antibodies. Nuclear (N) and cytosol (C) extracts were prepared from colon tissues on the last day of AOM/DSS-treated experiments. Nucleolin, α-tubulin, and β-actin were internal control; (**B**) NF-κB-related proteins were determined by Western blot analysis using specific antibodies. Relative ratio level was determined by densitometric analysis (Bio-rad Quantity One^®^ Software) normalized to β-actin. Values represent mean ± S.D. of three independent experiments. ^##^ *p* < 0.01, ^###^ *p* < 0.001 vs. the control group; * *p* < 0.05, ** *p* < 0.01 and *** *p* < 0.001 vs. the AOM/DSS group; significances between treated groups were determined using ANOVA and Dunnett’s post hoc test.

As the key components of inflammation include primary inflammatory cytokines, hematopoietic growth factors, and the master transcription factor NF-κB [14], we examined whether NF-κB was inhibited by AG in AOM/DSS-treated mice. As shown in Figure 6A, our results indicated that AG inhibited the nuclear translocation of NF-κB p65 protein in AOM/DSS-treated mice. We also found that AG inhibited the phosphorylation of IκBα and recovered the downregulated expression of IκBα. As NF-κB activation is important for the development of CAC, we next evaluated the expression of NF-κB-related proteins. AG and 5-ASA decreased the expression of NF-κB-related pro-survival genes, including Bcl-2, XIAP, and survivin (Figure 6B).

## 4. Discussion

CRC is one of the major causes of cancer-related deaths and poses a serious threat to human health [15]. Epidemiological and experimental studies suggest that environmental and dietary factors, including a diet low in fruits, fiber, and vegetables, and high in animal fat and other high-calorie foods, contribute to the development of CRC [16]. Furthermore, it is well known that chronic inflammation plays a critical role in colon carcinogenesis, with chronic IBD, such as ulcerative colitis and Crohn’s disease, markedly increasing the risk of the development of CAC [17].

During inflammation process, pro-inflammatory cytokines which are released by immune and non-immune cells may influence carcinogenesis [18]. It has been proposed that these cytokines contribute to carcinogenesis by influencing the survival, growth, mutation, proliferation, differentiation, and movement of tumor and stromal cells and by regulating angiogenesis [13]. The activities of iNOS and COX-2 has been shown to be associated with colorectal cancer. COX-2 is upregulated protein responsible for the overproduction of prostaglandins in inflammation contributing to the progression of IBD. iNOS activation lead to excessive production of NO, which causes DNA damage and inhibits the DNA repair process [19]. Indeed, in chronic inflammation, NO stimulates COX-2 activity, and increases p53 mutations, contributing to clone cellular expansion and genomic instability [20]. In our study, AOM/DSS-treated CAC mice fed AG (25 mg/kg/day; p.o.) produced lower amounts of IL-1β, IL-6, and TNF-α cytokines and had reduced iNOS and COX-2 protein expression. These data indicated that AG can exert an anti-inflammatory effect on CAC development by reducing inflammatory mediator production and protein expression.

These pro-inflammatory mediators are modulated by transcription factors including NF-κB. NF-κB subsequently controls the expression of genes that are key regulators of many physiological processes. These include the innate and adaptive immune responses, apoptosis, cell proliferation, inflammatory responses, and malignant transformation [21]. As NF-κB is suspected to act a key role in the process of inflammation and carcinogenesis, this could be a target of treatment for CRC [22]. Although the NF-κB transcription protein is associated exclusively with immunity and inflammation, it has also been proven that this transcription factor has an essential role in epithelial tissues, such as coordinating antibacterial immunity and maintaining a barrier function in the gastrointestinal system [23]. In unstimulated cells, NF-κB is remained in cytoplasm with inactivated state, which complexes with the inhibitory molecules called IκB proteins. However, activated NF-κB is located in the nucleus where it binds to specific DNA sequences called response elements and regulates the transcription of target genes. Based on these reports, we discovered that AG inhibited the NF-κB activation via the suppression of NF-κB p65 nuclear localization, phosphorylation, and degradation of IκBα in AOM/DSS-exposure CAC animal models. Furthermore, our findings revealed that the expression of NF-κB-associated anti-apoptotic proteins, including Bcl-2, XIAP, and survivin, decreased when the CAC-induced mice were treated with AG. NF-κB regulates the anti-apoptotic genes, for example, the inhibitor of Bcl-2 family, and apoptosis proteins (IAPs), whose products prevent cell death [24]. Among the Bcl-2 gene family, the prosurvival members are regulated by NF-κB activation that conferred protection against hypoxia and nitric oxide-induced injury in primary hippocampal neurons as well as its ability to promote the survival of peripheral B cells in response to c-Rel and RelA [25]. IAPs were transcriptionally activated by NF-κB, leading to the amplification of the positive feedback mechanism of NF-κB activation. This resulted in the enhancement of its anti-apoptotic function. XIAP attenuates the caspase activation and has been implicated in NF-kB-mediated suppression of JNK signaling [26]. In addition, survivin is the smallest member of the mammalian IAP family, which is expressed in most human tumors [27]. Overexpression of survivin in tumors is generally associated with poor prognosis and drug resistance [28]. Based on these mentioned reports, we speculated that AG might inhibit the proliferation of tumor cells via downregulation of the NF-κB-associated proteins.

## 5. Conclusions

In the present study, AG supplementations effectively suppressed inflammation-associated colon carcinogenesis in AOM/DSS-treated mice. Our findings indicated that AG decreased the levels of inflammatory enzymes such as iNOS and COX-2 and the production of inflammatory cytokines via regulation of NF-κB activation and downregulation of NF-κB-mediated proteins. Taken together, our findings indicated that the chemopreventive effects of AG against colon carcinogenesis were closely associated with anti-inflammatory mechanisms. AG is thus a promising natural preventive agent against CAC with the potential to help maintain human health through dietary supplementation.
